# Compensation for Electrode Detachment in Electrical Impedance Tomography with Wearable Textile Electrodes

**DOI:** 10.3390/s22249575

**Published:** 2022-12-07

**Authors:** Chang-Lin Hu, Zong-Yan Lin, Shu-Yun Hu, I-Cheng Cheng, Chih-Hsien Huang, Yu-Hao Li, Chien-Ju Li, Chii-Wann Lin

**Affiliations:** 1 Industrial Technology Research Institute, Hsinchu 310, Taiwan; 2 Biomedical Electronics and Bioinformatics, National Taiwan University, Taipei 106, Taiwan; 3 College of Law, National University of Kaohsiung, Kaohsiung 811, Taiwan; 4 Department of Electrical Engineering, National Tsing Hua University, Hsinchu 300, Taiwan; 5 Department of Electrical Engineering, National Cheng Kung University, Tainan 701, Taiwan; 6 Department of Biomedical Engineering, National Taiwan University, Taipei 106, Taiwan

**Keywords:** compensation, detached electrode, wearable textile electrode, belt, EIT

## Abstract

Electrical impedance tomography (EIT) is a radiation-free and noninvasive medical image reconstruction technique in which a current is injected and the reflected voltage is received through electrodes. EIT electrodes require good connection with the skin for data acquisition and image reconstruction. However, detached electrodes are a common occurrence and cause measurement errors in EIT clinical applications. To address these issues, in this study, we proposed a method for detecting faulty electrodes using the differential voltage value of the detached electrode in an EIT system. Additionally, we proposed the voltage-replace and voltage-shift methods to compensate for invalid data from the faulty electrodes. In this study, we present the simulation, experimental, and in vivo chest results of our proposed methods to verify and evaluate the feasibility of this approach.

## 1. Introduction

Electrical impedance tomography (EIT) is a radiation-free and noninvasive medical image reconstruction technique, in which a current is injected and the reflected voltage is received through electrodes [1]. EIT can provide continuous, real-time, regional impedance distribution for long-term monitoring [2]. Thus, EIT has been widely used to monitor continuous lung aeration conditions at the bedside [3,4,5,6]. Furthermore, EIT has been regarded as a promising medical imaging technology for the chest, even though EIT systems have low spatial resolution [7]. In 1978, Henderson et al. used two-dimensional matrix electrodes fixed on the front of the human chest and a single large electrode fixed on the back to estimate the resistivity distribution of the thorax [8]. Brown et al. used 16 electrodes to transmit small currents into a pair of adjacent electrodes and then reconstructed the EIT image obtained using the back-projection method [9,10]. To generate a chest image, EIT systems inject small currents with a certain pattern through electrodes that attach to the body for electrical stimulation [11,12,13]. Hua et al. examined edema and apnea according to the resistivity of the lungs on an EIT image [14]. The calculated regional volume in an EIT image is viewed as a useful index of lung function [15]. Thus, EIT systems have been used to monitor patients receiving mechanical ventilation in hospitals [16,17].

Traditional EIT systems usually assess the internal electrical conductivity of the lungs using adhesive electrodes attached to the skin of the thorax [18]. However, adhesive electrodes can cause physical discomfort and then increase the risk of skin injuries, particularly during prolonged measurements [19]. Furthermore, the conductive gel can dry up after long-term usage, which will significantly impact the signal quality of the electrodes [20,21]. Additionally, studies have proposed the use of wearable health-monitoring systems to help individuals manage their health conditions more effectively and easily provide more health-related information to medical personnel [22,23,24,25,26,27]. Thus, several wearable EIT devices have been developed for lung-health monitoring [15,28,29,30,31]. Moreover, electrically conductive, fiber-based materials with high sensitivity have been developed in various types of healthcare clothing.

Sentec AG Switzerland has commercialized textile electrodes for EIT [32,33,34]. However, such textile electrodes require a conductive gel placed on the back of the belt to reduce skin contact impedance. Hu et al. used a portable EIT system with textile electrodes integrated into a clothing belt to monitor the lungs [35]. A dry, wearable textile electrode belt would not suffer from the problem of drying out after long-term usage because it does not need any conductive gel.

EIT reconstructs resistivity distributions inside the body by injecting a current and receiving the reflected voltage signal from the collected voltages using 16 electrodes attached to the surfaces of the body [36,37]. Therefore, EIT electrodes require good connection with the skin for data acquisition and image reconstruction. However, detached electrodes are a common occurrence and cause measurement errors in EIT clinical applications [38]. Several factors, such as patient body movement, inadvertent cable pull, and clinical staff inexperience in operating the device, can contribute to electrode disconnection [39]. Faulty electrodes would produce significant artifacts in the EIT images. Thus, some studies have proposed methods for detecting disconnected electrodes and compensating for the invalid data from faulty electrodes using a specific algorithm. Adler proposed a method for calculating the resistivity distributions using compensated data modified by the noise covariance matrix in the maximum a posteriori reconstruction algorithm from disconnected electrodes [38]. The EIT image is reconstructed by terms of a maximum a posteriori including the a priori estimates of image and measurement noise cross-correlations. Then, the faulty electrode data could be modelled as infinite noise on all measurement data by the affected electrodes. However, the method could not be applied in clinical settings because it requires a priori information of the disconnected electrodes. Asfaw et al. proposed a method for automatically detecting one erroneous electrode based on comparisons between simulated and measured voltages [40]. The method assumes that all valid measurement data are related to the EIT image reconstruction model, while the invalid measurement data from detached electrodes are unrelated. Then, the data of an electrode are estimated by the measurement data from all other electrodes and compared to the measurements. However, this method is not suitable for real-time applications because of its computational complexity. Hartinger et al. proposed an algorithm for the real-time management of disconnected electrodes by applying the principle of reciprocity [39]. The approach weights each measurement data in accordance with its compliance based on the principle of voltage–current reciprocity. However, the method still requires extra reciprocity measurement and increases computational costs.

To address these problems, in this study, we proposed a method for detecting faulty electrodes using the differential voltage value of the detached electrodes in EIT systems. Additionally, we proposed the voltage-replace and voltage-shift methods to compensate for invalid data from the faulty electrodes. Our proposed methods estimate the detached electrodes and compensate for invalid data based on the measured voltages. Because our proposed methods would not require a modified data acquisition scheme or a modified reconstruction algorithm, these methods have low computation complexity. In this study, we present the simulation, experimental, and in vivo chest results of our proposed methods to verify and evaluate the feasibility of this approach.

## 2. Materials and Methods

### 2.1. Image Reconstruction

In this study, we estimated the conductivity distribution by determining current stimulation patterns and boundary conditions using a forward model of EIT. The forward solutions could be computed using the finite-element method (FEM) by discretizing the domain. Triangular elements were chosen for use in the EIT model, and the edges of the elements are vertex nodes $\varphi_{i}$ representing certain nodal potential values in EIT [41]. The voltage value of each element was modeled with basis functions $N_{i}$ for per triangular element i. The potential on each element could be approximated as follows:

(1) $$\tilde{\varphi }=\overset{M}{\underset{i=1}{\sum }}\varphi_{i}N_{i}$$

where M is the number of vertex nodes and $\varphi_{i}$ is nodal potential values. The distribution of the electric potential per node could be expressed in the form of a matrix. The unknown potential could be expressed using the following linear equation [42]:

(2) Y(σ)q=k

where q is a vector of unknown nodal potentials, current electrode potentials, and voltage electrode potentials. k represents the collection of the injected current values. Y represents the admittance matrix depending on the conductivity (σ). The reconstruction algorithm uses a linearized method, and the forward model could be expressed as follows [43]:

(3) u=Hσ

where σ represents the conductivity. u is the voltage electrode potentials that are already known from the electrode measurements. H represents the Jacobian matrix, which expresses the voltage electrode potentials and background conductivity. In this study, the software package Electrical Impedance and Diffuse Optical Reconstruction Software (EIDORS), the meshing software NETGEN, and the reconstruction algorithm Graz Consensus Reconstruction Algorithm for EIT (GREIT) were used to simulate and reconstruct EIT images [44,45,46].

### 2.2. Detection of Faulty Electrodes

In this study, the bipolar adjacent stimulation pattern was used for our portable 16-electrode EIT system. Major changes in conductivity occur on the object boundary because of the intensity of the injection current in the EIT system [42]. Additionally, the injected current and induced voltage distribution are high near the boundary of the injection electrode pair. The induced voltage differences are calculated between the electrode pairs. A set of 256 data is measured at each current injection cycle, where the readings from all electrodes and the recordings from their nearest three electrodes are removed. Thus, the 208-voltage dataset is calculated to reconstruct the distribution of electrical conductivity [47]. According to a previous study, the values of voltage differences between adjacent electrode pairs would generate one positive and one negative spike when there is a detached electrode [35]. Voltage difference (ΔV) spikes in detached electrodes are more than 10 times larger than the values of ΔV in well-contacted electrodes. Therefore, we could easily and quickly detect which electrode has a bad connection with the system from the location of the voltage spikes. Additionally, the effect of the voltage difference when detached electrodes inject a current and are set as the ground has more impact than that when well-contacted electrodes inject a current. Thus, in this study, we only considered the effect of the voltage difference when detached electrodes inject a current and are set as the ground.

**Figure 1:**
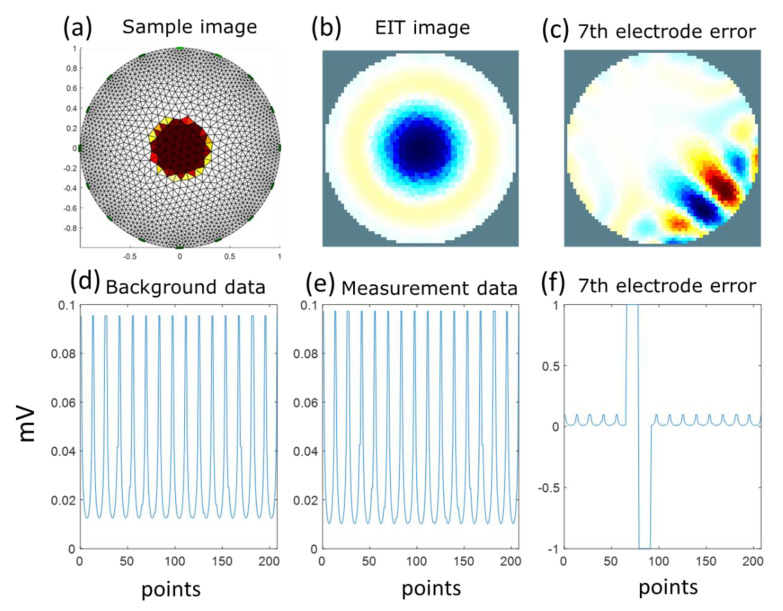
Simulated sample and reconstructed images. (**a**) Sample image. (**b**) EIT image. (**c**) EIT image with the seventh electrode detached. (**d**) Background data. (**e**) Measurement data with 16 well-contacted electrodes. (**f**) Measurement data with the seventh electrode detached.

One circular inclusion with low conductivity was simulated using EIDORS for a 16-electrode EIT system with an adjacent stimulation pattern, as shown in Figure 1 [44,48]. Figure 1a shows a two-dimensional circular sample image for testing image reconstruction under different conditions of electrode contacts. Figure 1d shows the voltage differences within the 208-voltage measurement dataset in the background conductivity without circular inclusion. Figure 1e shows the 208-voltage measurement dataset with circular inclusion. We simulated the 208-voltage measurement dataset when the seventh electrode was detached, as shown in Figure 1f. We found two spikes when the sixth and seventh electrodes inject a current. Figure 1b,c shows the EIT images reconstructed with 16 electrodes well contacted and the seventh electrode detached.

**Figure 2:**
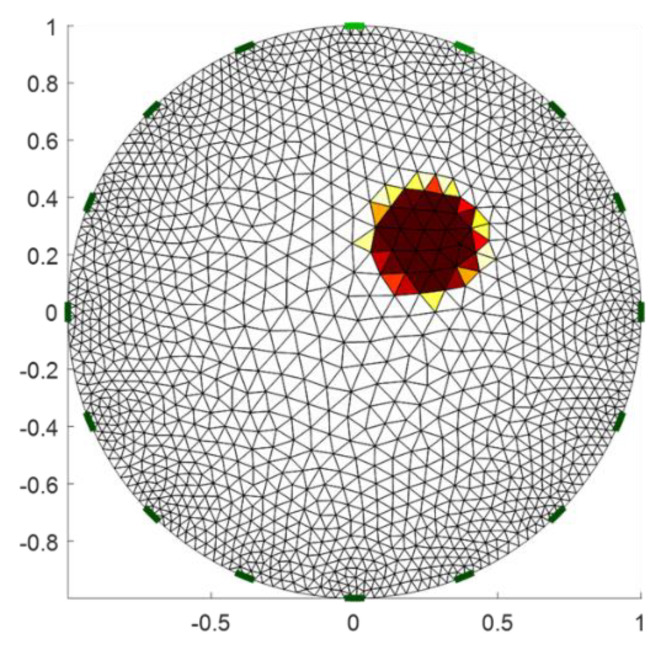
Simulated phantom sample.

**Figure 3:**
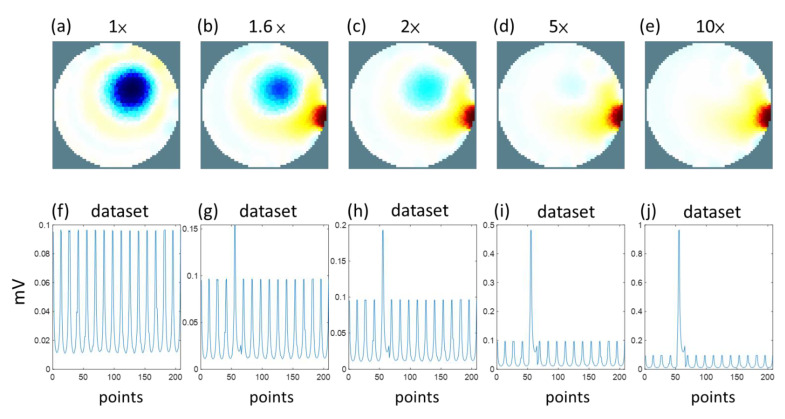
Simulated EIT images and the 208-voltage measurement dataset. (**a**) EIT image without spike value. (**b**) EIT image with spike value 1.6×. (**c**) EIT image with spike value 2×. (**d**) EIT image with spike value 5×. (**e**) EIT image with spike value 10×. (**f**) Measurement data with 16 well-contacted electrodes. (**g**) Measurement data with spike value 1.6×. (**h**) Measurement data with spike value 2×. (**i**) Measurement data with spike value 5×. (**j**) Measurement data with spike value 10×.

We simulated one sample image of a circular inclusion with low conductivity, as shown in Figure 2, and then reconstructed the EIT image using different levels of badly contacted electrodes. Here we only considered one voltage spike in the fifth electrode. Figure 3a shows the reconstructed EIT image using the 208-voltage measurement dataset shown in Figure 3f. Figure 3f–j displays the 208-voltage measurement dataset. Figure 3g–j shows that the spike values are 1.6 times, 2 times, 5 times, and 10 times larger than the values in Figure 3f at the area of the measurement data when the fifth electrode injected a current, respectively. Figure 3b–e shows the reconstructed EIT image using the 208-voltage measurement dataset shown in Figure 3g–j. We found that the EIT image of a circular inclusion gradually disappeared when the spike values increased from 1.6 times to 10 times. Therefore, we could determine the disconnected electrode using the 208-voltage measurement dataset when the spike value is 10 times larger than the mean value.

### 2.3. Algorithm for Compensating for the Invalid Data from Faulty Electrodes

We proposed two methods, the voltage-replace and voltage-shift methods, to compensate for spike data caused by the current injected by the detached electrode.

#### 2.3.1. Voltage-Replace Method

**Figure 4:**
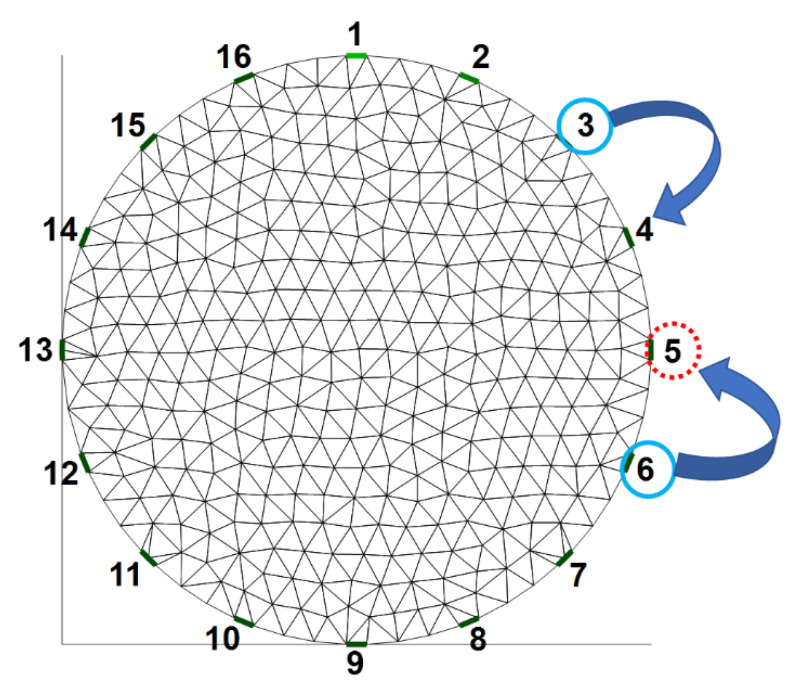
Illustration of voltage-replace method.

**Figure 5:**
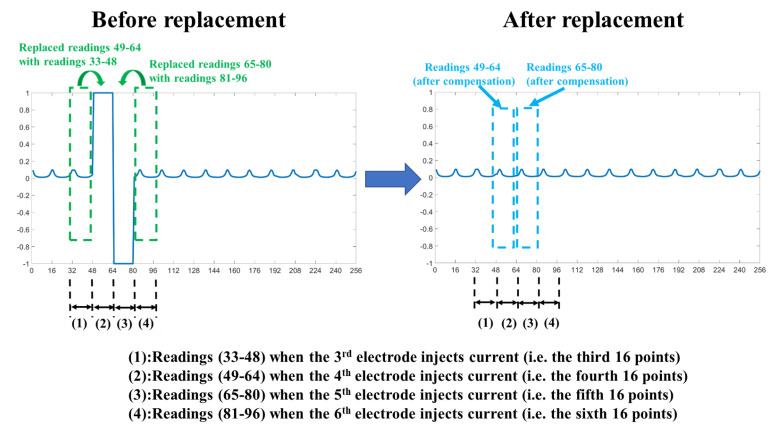
Illustration of compensation with voltage-replace method for invalid data.

The voltage changes in each electrode are correlated with the adjacent electrodes. Thus, we proposed the use of the voltage-replace method to use the measurement voltage values when the adjacent electrode injects a current to directly replace the measurement voltage values when the disconnected electrode injects a current and is set as the ground. In Figure 4, when the fifth electrode is disconnected from the system, there would be two sets of invalid data when the fourth and fifth electrodes inject a current. Thus, the measurement voltage values when the sixth electrode injects a current to directly replace the invalid data when the fifth electrode injects current. The measurement voltage values when the third electrode injects a current to directly replace the invalid data when the fourth electrode injects a current. When the fourth electrode injects a current, the fifth electrode is set as the ground at the same time. We showed more details of the voltage-replace method in Figure 5. A set of 256 data was measured at each current injection cycle. The first 16 points (i.e., from point 1 to point 16) are measured from 16 electrodes when the first electrode injects a current. The second 16 points (i.e., from point 17 to point 32) are measured from 16 electrodes when the second electrode injects a current. In this example, the fifth electrode is detached in the EIT system. Thus, the third 16 points (i.e., readings 33–48) replaced the fourth 16 points (i.e., readings 49–64) and the sixth 16 points (i.e., readings 81–96) replaced the fifth 16 points (i.e., readings 65–80), as shown in Figure 5.

Difference EIT calculates the conductivity distribution between the measured voltage values and the reference voltage values. Therefore, when we use the voltage-replace method to compensate for the invalid data from faulty electrodes, we must replace these data in the measured voltage data and the reference voltage data. Here, the reference voltage data indicate the background data acquired from a homogeneous medium in the acrylic phantom or unnecessary chest movements during full exhalation in human subjects.

#### 2.3.2. Voltage-Shift Method

**Figure 6:**
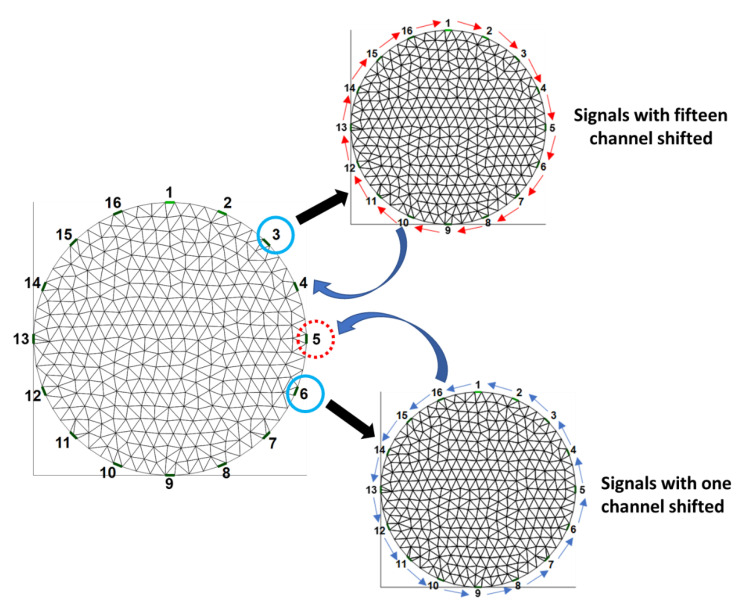
Illustration of voltage-shift method.

We proposed another voltage-shift method, which is the modified voltage-replace method, to compensate for invalid data due to the faulty electrode. In Figure 6, the fifth electrode is detached from the system. All of the 16 electrodes receive voltage signals when the sixth electrode injects a current, and then, the signals in all of the 16 channels are shifted by one electrode position to be similar to the shifted 16-channel signals from current injection of the fifth electrode. Furthermore, we used all of the shifted 16-channel signals to replace the invalid data when the first electrode injects a current. We applied the same approach to shift 15 electrode positions for all of the 16-channel signals when the third electrode injects a current. After that, we used all of the shifted 16-channel signals from the current injection of the third electrode to replace the invalid data when the fourth electrode injects a current. When the fourth electrode injects a current, the fifth electrode is set as the ground at the same time.

In this example, the fifth electrode is detached in the EIT system. In the voltage-shift method, the third 16 points (i.e., readings 33–48) are shifted by one point. Thus, the value of point 33 is shifted to point 34 and the value of point 48 is shifted to point 33. The sixth 16 points (i.e., readings 81–96) are shifted fifteen points. Thus, the value of point 81 is shifted to point 80 and the value of point 96 is shifted to point 95. Finally, the third 16 points (i.e., readings 33–48) that were shifted one point replaced the fourth 16 points (i.e., readings 49–64) and the sixth 16 points (i.e., readings 81–96) that were shifted fifteen points replaced the fifth 16 points (i.e., readings 65–80).

### 2.4. Portable EIT System

**Figure 7:**
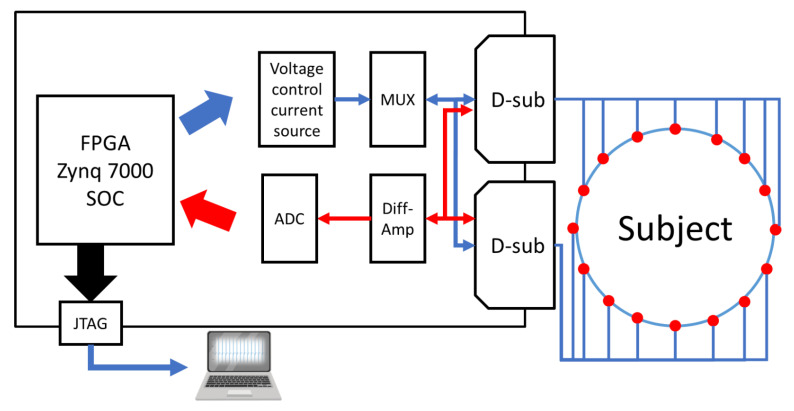
Block diagram of the portable EIT system.

Figure 7 shows a block diagram of the portable EIT system. The hardware consists of the transmitting current part, the receiving signal part, and a user interface. The phantom or human subjects under testing are connected to the multiplexers (DG408, MAXIM) of the EIT system to switch the 16 surface electrodes. The EIT system is based on the Xilinx Zynq-7000 SoC, providing a combination of Advanced RISC Machine processors with microcontroller peripherals. The excitation current is generated by the Howland voltage-controlled current source (VCCS) circuit with a 16-bit digital-to-analog converter (DAC8820, TI). The DAC output analog voltage was from 0 V to +1 V at frequencies ranging from approximately 25 kHz to 200 kHz. Here, the VCCS delivers a constant current of 5 mA at 100 kHz. After that, the multiplexer was designed to switch the current injection path to the selected electrode.

In the receiving signal part, 16 amplifiers (INA128, TI) were used to measure differential voltages between two adjacent electrodes (electrodes 1 and 2, electrodes 2 and 3, …, electrodes 16 and 1). The output voltages of the differential amplifiers were digitized by applying them to a 12-bit and 1-volt analog-to-digital converter ADC (AFE5851, TI). In this study, the Zynq-based board was used to communicate with MATLAB software using the JTAG interface.

### 2.5. Simulation, Experimental, and In Vivo Lung Setups

We used EIDORS to simulate a cylinder tank model containing 16 electrodes with adjacent injection patterns. One acrylic rod with a diameter of 4 cm was simulated and placed on three locations at (x = 2.5 cm, y = −5.5 cm), (x = −2.5 cm, y = 5.5 cm), and (x = −2.5 cm, y = −5.5 cm). We reconstructed 3 EIT images using 16 electrodes with the first, seventh, and fifth electrodes detached, respectively. Then, we compensated for the invalid data using our proposed voltage-replace and voltage-shift methods to reconstruct EIT images again.

**Figure 8:**
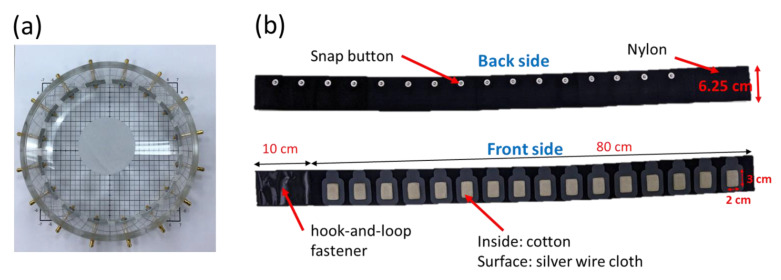
A cylinder acrylic tank and a wearable textile electrode belt. (**a**) An acrylic rod was placed in the acrylic tank. (**b**) Both sides of the wearable textile electrode belt.

Additionally, our experimental setup used a cylindrical acrylic tank (14 cm in diameter) filled with conductive saline, and the tank was mounted on one layer of 16 EIT electrodes (Figure 8a). One acrylic rod with a diameter of 4 cm was located at three locations: (x = 2.5 cm, y = −5.5 cm), (x = −2.5 cm, y = 5.5 cm), and (x = −2.5 cm, y = −5.5 cm). Our designs to compensate for the invalid data from the detached electrodes in our experimental setup were the same as those for the EIDORS simulation.

The electrical conductivity distribution of acrylic rod imaging was reconstructed from the background and measurement data. Here, the background data indicate the received signal coming from homogeneous scattering mediums only in the acrylic tank, and the measurement data indicate the received voltages from the acrylic rod placed in the acrylic tank.

We evaluated the figures of merit of the EIT image and presented the potential for performance improvements after compensating for invalid data using our proposed methods. We used position error (PE) and shape deformation (SD) to quantitatively analyze the reconstructed EIT images from the simulation and experimental data [46,49]. We computed the PE as the mismatch between the detected and actual positions.

(4) $$\mathrm{PE}\;=\left|r_{D}-r_{R}\;\right|$$

where $r_{D}$ is the detected position of the target EIT images and $r_{R}$ represents the position of the actual object. We calculated the SD by dividing the difference between the detected and actual sizes by the size of the actual object.

(5) $$\mathrm{SD}=\left|\frac{A_{D}-A_{R}}{A_{R}}\right|$$

where $A_{D}$ is the detected size of the EIT image and $A_{R}$ represents the size of the actual object.

### 2.6. In Vivo Lung Imaging with Wearable Textile Electrode Belt

The in vivo EIT data of the chest in healthy volunteers were acquired using the wearable textile electrode belt shown in Figure 8b. This experiment was performed according with the World Medical Association Declaration of Helsinki on Ethical Principles for Medical Research Involving Human Subjects [50,51]. Informed consent was obtained from the healthy volunteer. The textile electrode belt was wrapped around the chest of the human body. Figure 8b presents the textile electrode belt. The elastic band was made of nylon, and 16 snap buttons were attached to the back of the belt. Additionally, the 16 textile electrodes were stitched to the elastic band in the front of the belt. We recorded the in vivo data from one completed breathing cycle and acquired five lung EIT images for this experiment.

Here, we reconstructed lung images using difference EIT and calculated the conductivity distribution between the reference and measurement voltage values. Thus, we asked the volunteers to fully exhale to record the background data. Then, the background data were processed to a 208-voltage dataset and served as the reference voltage values. The measurement data were collected during natural breathing. After that, the measurement data were processed to a 208-voltage dataset and served as the measurement voltage values.

We used the structural similarity index measurement (SSIM) to quantitatively analyze similarities in brightness, contrast, and structure between the original EIT lung image and the faulty EIT lung image or the compensated EIT lung image [52]. The SSIM is used to measure the similarity between two images. Higher SSIM values indicate that the two images have smaller relative image error and are similar.

(6) $$SSIM\left(x,y\right)=\frac{\left(2\mu_{x}\mu_{y}+C1\right)}{\left(\mu_{x}^{2}+\mu_{y}^{2}+C1\right)}\frac{\left(2\sigma_{xy}+C2\right)}{\left(\sigma_{x}^{2}+\sigma_{y}^{2}+C2\right)}$$

where $\mu_{x}$ and $\mu_{y}$ represent the mean values of images x and y, respectively. $\sigma_{x}$ and $\sigma_{y}$ are the standard deviations of images x and y. C1 and C2 represent constants set to avoid the denominator being zero.

### 2.7. The EIT System with Compensation for Invalid Data

**Figure 9:**
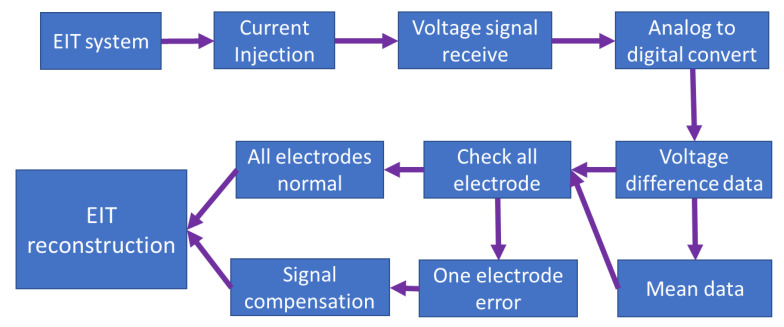
A block diagram of the 16 electrodes EIT system with compensation for invalid data.

A block diagram of the 16-electrode EIT system with compensation for invalid data is presented in Figure 9. The current was generated by the VCCS circuit, and the reflected voltage was received through the 16 electrodes. The received voltages were digitized using a 12-bit analog-to-digital converter. Furthermore, we could calculate and record the voltage differences between a pair of electrodes in the EIT system. After that, we calculated the mean value of the 16 sets of voltage difference data. We could determine which electrode is detached if the spike value is 10 times larger than the mean value. If one electrode is disconnected from the EIT system, we could compensate for the invalid data using the voltage-replace and voltage-shift methods to replace the spike data caused by the current injected by the detached electrode.

## 3. Results and Discussion

### 3.1. EIDORS Simulation

**Figure 10:**
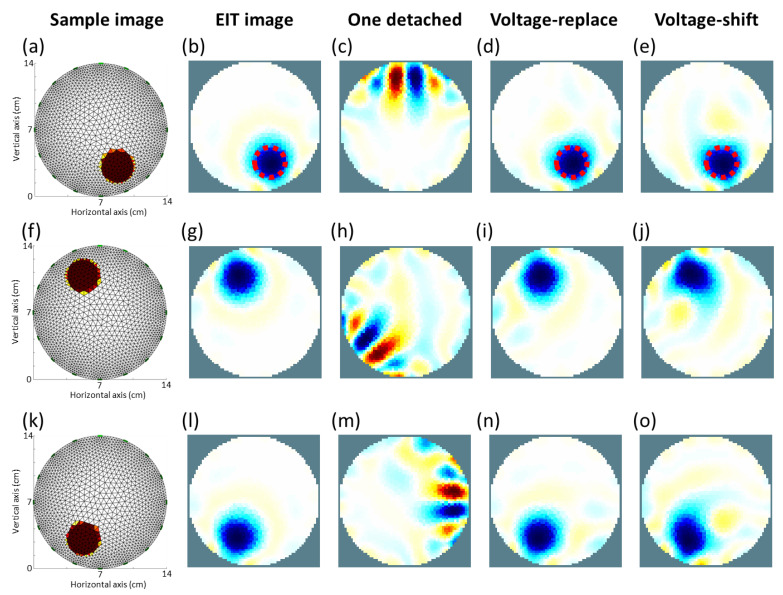
Simulated electrical impedance tomography (EIT) images. (**a**) Sample image with circular inclusion at (x = 2.5 cm, y = −5.5 cm). (**b**) EIT image of (**a**). (**c**) EIT image of (**a**) with first electrode detached. (**d**) Image reconstruction for (**c**) using the voltage-replace method. (**e**) Image reconstruction for (**c**) using the voltage-shift method. (**f**) Sample image with circular inclusion at (x = −2.5 cm, y = 5.5 cm). (**g**) EIT image of (**f**). (**h**) EIT image of (**f**) with seventh electrode detached. (**i**) Image reconstruction for (**h**) using the voltage-replace method. (**j**) Image reconstruction (**h**) using the voltage-shift method. (**k**) Sample image with circular inclusion at (x = −2.5 cm, y = −5.5 cm). (**l**) EIT image of (**k**). (**m**) EIT image of (**k**) with the fifth electrode detached. (**n**) Image reconstruction for (**m**) using the voltage-replace method. (**o**) Image reconstruction for (**m**) using the voltage-shift method.

Figure 10a,f,k shows that one circular inclusion with low electrical conductivity was placed at three positions of the cylinder tank using the 2D FEM method. Figure 10b,g,l shows the simulated EIT images in Figure 10a,f,k in the cylinder tank. Figure 10c shows the reconstructed EIT image of Figure 10a using 16 electrodes with the first electrode detached. Figure 10d,e shows the re-reconstructed EIT images of Figure 10a with the first electrode detached using the voltage-replace and voltage-shift methods, respectively. Figure 10h shows the reconstructed EIT image of Figure 10f using 16 electrodes with the seventh electrode detached. Figure 10i,j shows the re-reconstructed EIT images of Figure 10f with the eleventh electrode detached using the voltage-replace and voltage-shift methods, respectively. Figure 10m shows the reconstructed EIT image of Figure 10k using 16 electrodes with the fifth electrode detached. Figure 10n,o shows the re-reconstructed EIT images of Figure 10k with the fifth electrode detached using the voltage-replace and voltage-shift methods, respectively.

Table 1 lists the SD and PE values of the three kinds of EIT images (original, voltage-replace method, and voltage-shift method images) obtained from EIDORS simulation results. In the results of the first circular inclusion shown in Figure 10a, the SD and PE values were 11.3% and 0.3 mm for the EIT image with 16 well-contacted electrodes, 12.19% and 1 mm for the EIT image with compensation using the voltage-replace method, and 15.6% and 2.3 mm for the EIT image with compensation using the voltage-shift method. In the results of the second circular inclusion shown in Figure 10f, the SD and PE values were 10.37% and 0.04 mm for the EIT image with 16 well-contacted electrodes, 12.87% and 1 mm for the EIT image with compensation using the voltage-replace method, and 22.8% and 2.3 mm for the EIT image with compensation using the voltage-shift method. In the results of the second circular inclusion shown in Figure 10k, the SD and PE values were 12.63% and 0. 5 mm for the EIT image with 16 well-contacted electrodes, 13.45% and 1.2 mm for the EIT image with compensation using the voltage-replace method, and 21.27% and 1.5 mm for the EIT image with compensation using the voltage-shift method. We found that by using the voltage-replace method, the EIT images will have better quality than those using the voltage-shift method. Although the EIT images with compensation using the voltage-shift method have little distortion, the results of the three methods for reconstructing EIT images were similar.

### 3.2. Experimental Results

**Figure 11:**
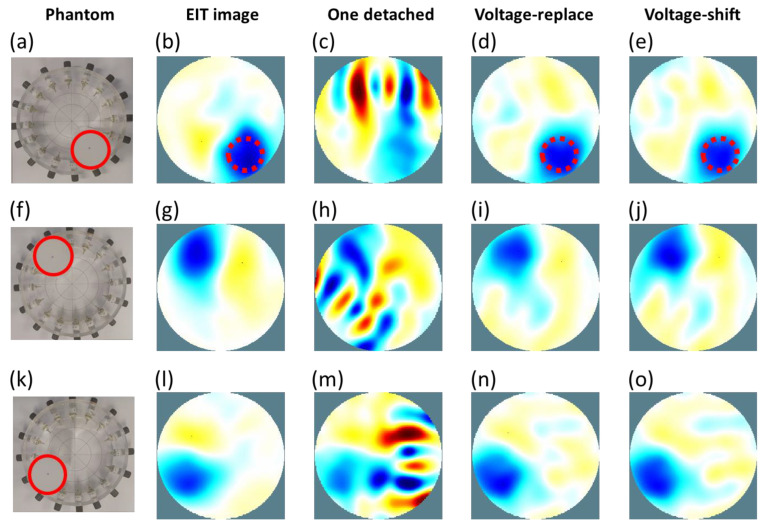
Experimental electrical impedance tomography (EIT) images. (**a**) Sample image with a circular inclusion at (x = 2.5 cm, y = −5.5 cm). (**b**) EIT image of (**a**). (**c**) EIT image of (**a**) with the first electrode detached. (**d**) Image reconstruction for (**c**) using the voltage-replace method. (**e**) Image reconstruction for (**c**) using the voltage-shift method. (**f**) Sample image with a circular inclusion at (x = −2.5 cm, y = 5.5 cm). (**g**) EIT image of (**f**). (**h**) EIT image of (**f**) with the seventh electrode detached. (**i**) Image reconstruction for (**h**) using the voltage-replace method. (**j**) Image reconstruction for (**h**) using the voltage-shift method. (**k**) Sample image with a circular inclusion at (x = −2.5 cm, y = −5.5 cm). (**l**) EIT image of (**k**). (**m**) EIT image of (**k**) with the fifth electrode detached. (**n**) Image reconstruction for (**m**) using the voltage-replace method. (**o**) Image reconstruction for (**m**) using the voltage-shift method.

Figure 11a,f,k shows that one acrylic rod was placed at three positions in the cylinder tank. Figure 11b,g,l shows the reconstructed EIT images of Figure 11a,f,k in the cylinder tank. Figure 11c shows the EIT image of Figure 11a using 16 electrodes with the first electrode detached. Figure 11d,e shows the re-reconstructed EIT images of Figure 11a with the first electrode detached using the voltage-replace and voltage-shift methods, respectively. Figure 11h shows the reconstructed EIT image of Figure 11f using 16 electrodes with the eleventh electrode detached. Figure 11i,j shows the re-reconstructed EIT images of Figure 11f with the eleventh electrode detached using the voltage-replace and voltage-shift methods, respectively. Figure 11m shows the reconstructed EIT image of Figure 11k using 16 electrodes with the fifth electrode detached. Figure 11n,o shows the re-reconstructed EIT images of Figure 11k with the fifth electrode detached using the voltage-replace and voltage-shift methods, respectively.

Table 2 lists the SD and PE values of the three kinds of EIT images (original, voltage-replace method, and voltage-shift methods) obtained with experimental results. In the results of the acrylic rod shown in Figure 11a, the SD and PE values were 18.77% and 0.5 mm for the EIT image with 16 well-contacted electrodes, 20.2% and 1.7 mm for the EIT image with compensation using the voltage-replace method, and 19.13% and 1.6 mm for the EIT image with compensation using the voltage-shift method. In the results of the second circular inclusion shown in Figure 11f, the SD and PE values were 20.08% and 0.7 mm for the EIT image with 16 well-contacted electrodes, 20.75% and 1.62 mm for the EIT image with compensation using the voltage-replace method, and 21.18% and 1.72 mm for the EIT image with compensation using the voltage-shift method. In the results of the second circular inclusion shown in Figure 11k, the SD and PE values were 21.28% and 1.21 mm for the EIT image with 16 well-contacted electrodes, 22.33% and 1.81 mm for the EIT image with compensation using the voltage-replace method, and 22.71% and 1.92 mm for the EIT image with compensation using the voltage-shift method.

We found that the results of the EIT images obtained from the EIDORS simulation (Figure 10) were similar to those obtained from phantom experiments (Figure 11). When there was one electrode disconnected from the EIT system, the detached electrode would produce significant artifacts in the EIT images shown in Figure 10c,h,m and Figure 11c,h,m. After the compensation for the invalid data using the voltage-replace method, the signification artifact was removed and the original EIT images shown in Figure 10d,i,n and Figure 11d,i,n were recovered. Additionally, after the compensation for the invalid data using the voltage-shift method, the faulty EIT images were significantly improved, as shown in Figure 10e,j,o and Figure 11e,j,o. All PE values were similar between the original and compensated EIT images, as shown in Table 1 and Table 2. The SD values of the compensated EIT images were closer to those of the original EIT images in the experimental results (Table 2) than those in the simulation results (Table 1). This proves that the experimental imaging using the proposed methods corresponded to the theoretical imaging from the simulation using the proposed methods.

### 3.3. Results of In Vivo Lung EIT Data

**Figure 12:**
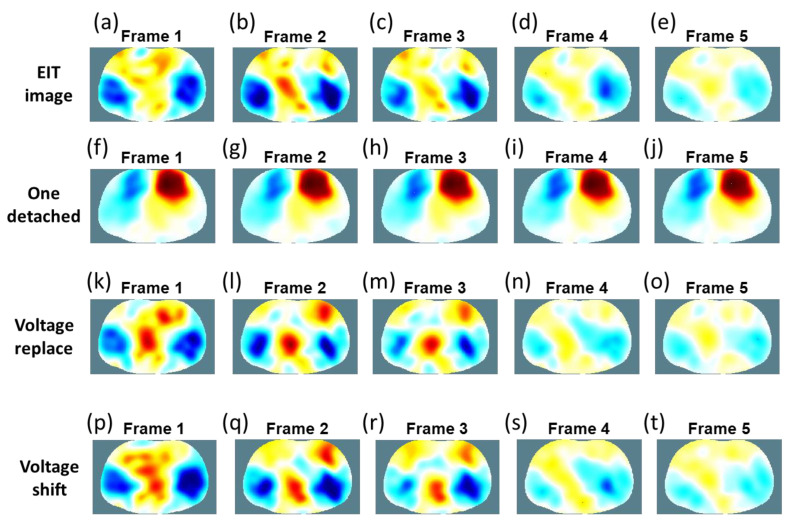
The EIT images of the chest over one breathing cycle. (**a**) Frame 1 of the EIT image. (**b**) Frame 2 of the EIT image. (**c**) Frame 3 of the EIT image. (**d**) Frame 4 of the EIT image. (**e**) Frame 5 of the EIT image. (**f**) Frame 1 of the faulty image. (**g**) Frame 2 of the faulty image. (**h**) Frame 3 of the faulty image. (**i**) Frame 4 of the faulty image. (**j**) Frame 5 of the faulty image. (**k**) Frame 1 using the voltage-replace method. (**l**) Frame 2 using the voltage-replace method. (**m**) Frame 3 using the voltage-replace method. (**n**) Frame 4 using the voltage-replace method. (**o**) Frame 5 using the voltage-replace method. (**p**) Frame 1 using the voltage-shift method. (**q**) Frame 2 using the voltage-shift method. (**r**) Frame 3 using the voltage-shift method. (**s**) Frame 4 using the voltage-shift method. (**t**) Frame 5 using the voltage-shift method.

The EIT images of the chest (i.e., from frames 1–5) over one breathing cycle using a portable EIT system with the wearable textile electrode belt are shown in Figure 12a–e. In this study, our goal was to verify the feasibility of our proposed compensation algorithm in EIT lung imaging that uses wearable textile electrodes with our proposed potable EIT system. Thus, we picked one frame per second in EIT lung images to easily evaluate the results using the voltage-replace and voltage-shift methods from one completed breathing cycle. Each frame of an EIT image shows the conductivity distribution of the chest and consists of a matrix of 129 × 77 pixels (Figure 12). Figure 12a (frame 1) represents inhalation and Figure 12e (frame 5) represents exhalation. The changes in the impedance distribution of pulmonary ventilation from frames 1–5 were represented in the corresponding pixels of the image using different color tones determined by their magnitude of electrical conductivity. The largest value was shown in dark red, and the smallest value was shown in dark blue. The blue zones of both sides in such a functional EIT lung image represent the ventilated lung regions [53]. The larger and darker blue areas represent inhalation. In contrast, the smaller and lighter blue areas represent exhalation.

Figure 12f–j shows the reconstructed EIT lung images using the wearable textile electrode belt with the first electrode detached. Figure 12f (frame 1) represents the inhalation lung image and Figure 12i (frame 5) represents the exhalation lung image. However, we could not find any conductivity changes when the first electrode was detached. Thus, the faulty electrode would produce significant artifacts in EIT lung imaging. Figure 12k–o shows the re-reconstructed EIT lung images of Figure 12f–j with the first electrode detached using the voltage-replace method. Figure 12p–t shows the re-reconstructed EIT lung images of Figure 12f–j with the first electrode detached using the voltage-shift method. The voltage-replace and voltage-shift methods would remove the significant artifacts from the detached electrode and recover the original EIT lung image. Here, our proposed methods were applied to in vivo lung data with a wearable textile electrode belt because the wearable textile electrodes are suitable for long-term EIT monitoring. In addition, poor electrode contact with wearable textile electrodes is a more common occurrence and cause measurement errors in EIT applications during prolonged measurements. Furthermore, our proposed methods could be used in any kind of electrodes for EIT systems.

Table 3 shows the SSIM values of all images between the original EIT lung images with well-contacted electrodes and those under three conditions (i.e., one electrode detached, the voltage-replace method, and voltage-shift method). The SSIM values between the original EIT lung images and those with the first electrode detached are 0.5213, 0.5706, 0.6225, 0.6924, and 0.7158 from frames 1–5, respectively. The SSIM values between the original EIT lung images and those with the voltage-replace method are 0.8847, 0.8621, 0.8958, 0.9633, and 0.7873 from frames 1–5, respectively. The SSIM values between the original EIT lung images and those with the voltage-shift method are 0.9031, 0.9041, 0.9288, 0.9703, and 0.9883 from frames 1–5, respectively. The voltage-replace and voltage-shift methods would improve the image quality with the faulty electrode. These large conductivity changes occur on the boundary of the chest because of the intensity of the injection current in the EIT system [42]. Therefore, the injection current and reflected voltage distribution are high near the boundary of the injection electrode pair. We found that by using the voltage-shift method, the EIT image had better quality than that produced by using the voltage-replace method for in vivo lung EIT data. This is because the measurements related to the detached electrode from the voltage-shift method is closer to the ordinary mechanism. For instance, when the fifth electrode was detached, the voltage-replace method would copy measurements 33–48 to measurements 49–64 and copy measurements 81–96 to measurements 65–80. However, the voltage-shift method would complete a circular shift to the right for measurements 33–48 before copying to measurements 49–64 and complete a circular shift to the left for measurements 81–96 before copying to measurements 65–80. Hence, the important information from the measurement electrodes would be shifted one step closer to the detached electrode. In this study, we used the voltage-replace and voltage-shift methods to compensate for the invalid data with a single detached electrode in the EIT system. In future work, our proposed methods could be extended to handle multiple detached electrodes.

## 4. Conclusions

In this study, we proposed a method for detecting faulty electrodes using the differential voltage value of the detached electrode in EIT systems. Additionally, we have verified the validity of our proposed voltage-replace and voltage-shift methods to compensate for the invalid data when an electrode of an EIT system is detached in the simulation and experimental results. Furthermore, our proposed methods were applied to in vivo lung data using a portable EIT system with a wearable textile electrode belt. The in vivo results have verified the feasibility of our proposed methods.

## Figures and Tables

**Table 1:** Shape deformation (SD) and position error (PE) of the EIT images obtained with simulation, as shown in Figure 10.

	Circular Inclusion Shown in Figure 10a			Circular Inclusion Shown in Figure 10f			Circular Inclusion Shown in Figure 10k		
	EIT Image	Voltage-Replace	Voltage-Shift	EIT Image	Voltage-Replace	Voltage-Shift	EIT Image	Voltage-Replace	Voltage-Shift
SD (%)	11.30	12.19	15.60	10.37	12.87	22.80	12.63	13.45	21.27
PE (mm)	0.30	1.00	2.30	0. 40	1.00	2.30	0. 50	1.20	1.50

**Table 2:** Shape deformation (SD) and position error (PE) values of the EIT images, as shown in Figure 11.

	Circular Inclusion Shown in Figure 11a			Circular Inclusion Shown in Figure 11f			Circular Inclusion Shown in Figure 11k		
	EIT Image	Voltage-Replace	Voltage-Shift	EIT Image	Voltage-Replace	Voltage-Shift	EIT Image	Voltage-Replace	Voltage-Shift
SD (%)	18.77	20.20	19.13	20.08	20.75	21.18	21.28	22.33	22.71
PE (mm)	0.50	1.70	1.60	0.70	1.62	1.72	1.21	1.81	1.92

**Table 3:** Structural similarity index measurement (SSIM) values of all images between the original EIT lung images with well-contacted electrodes and those under the three conditions (i.e., one electrode detached, the voltage-replace method, and voltage-shift method), as shown in Figure 12.

	Original EIT Image				
SSIM	Frame 1	Frame 2	Frame 3	Frame 4	Frame 5
One detached	0.5213	0.5706	0.6225	0.6924	0.7158
Voltage-replace method	0.8847	0.8621	0.8958	0.9633	0.9873
Voltage-shift Method	0.9031	0.9041	0.9288	0.9703	0.9883

## Data Availability

Data can be made available upon request.
